# Chondrogenic medium in combination with a c-Jun N-terminal kinase inhibitor mediates engineered cartilage regeneration by regulating matrix metabolism and cell proliferation

**DOI:** 10.1093/rb/rbad079

**Published:** 2023-09-07

**Authors:** Peiling Zhang, Qianyi Wang, Jie Chen, Zheng Ci, Wei Zhang, Yu Liu, Xiaoyun Wang, Guangdong Zhou

**Affiliations:** Department of Plastic and Reconstructive Surgery, Shanghai Key Laboratory of Tissue Engineering, Shanghai Ninth People’s Hospital, Shanghai Jiao Tong University School of Medicine, Shanghai, 200023, China; National Tissue Engineering Center of China, Shanghai, 200241, China; National Tissue Engineering Center of China, Shanghai, 200241, China; Department of Research Institute of Plastic Surgery, Wei Fang Medical College, Wei Fang, Shandong, 261041, China; National Tissue Engineering Center of China, Shanghai, 200241, China; Department of Anesthesiology, Shanghai Ninth People’s Hospital, Shanghai Jiao Tong University School of Medicine, Shanghai, 200023, China; National Tissue Engineering Center of China, Shanghai, 200241, China; Department of Plastic and Reconstructive Surgery, Shanghai Key Laboratory of Tissue Engineering, Shanghai Ninth People’s Hospital, Shanghai Jiao Tong University School of Medicine, Shanghai, 200023, China; National Tissue Engineering Center of China, Shanghai, 200241, China; Department of Research Institute of Plastic Surgery, Wei Fang Medical College, Wei Fang, Shandong, 261041, China; National Tissue Engineering Center of China, Shanghai, 200241, China; Department of Research Institute of Plastic Surgery, Wei Fang Medical College, Wei Fang, Shandong, 261041, China; Department of Plastic Surgery, Tong Ren Hospital, Shanghai Jiao Tong University School of Medicine, Shanghai, 200050, China; Department of Plastic and Reconstructive Surgery, Shanghai Key Laboratory of Tissue Engineering, Shanghai Ninth People’s Hospital, Shanghai Jiao Tong University School of Medicine, Shanghai, 200023, China; National Tissue Engineering Center of China, Shanghai, 200241, China; Department of Research Institute of Plastic Surgery, Wei Fang Medical College, Wei Fang, Shandong, 261041, China

**Keywords:** JNK inhibitor, chondrogenic medium, in vitro, in vivo, cartilage regeneration

## Abstract

Cartilage tissue engineering is a promising strategy for repairing cartilage defects. However, achieving satisfactory cartilage regeneration *in vitro* and maintaining its stability *in vivo* remains a challenge. The key to achieving this goal is establishing an efficient cartilage regeneration culture system to retain sufficient active cells with physiological functions, generate abundant cartilage extracellular matrix (ECM) and maintain a low level of cartilage ECM degradation. The current chondrogenic medium (CM) can effectively promote cartilage ECM production; however, it has a negative effect on cell proliferation. Meanwhile, the specific c-Jun N-terminal kinase pathway inhibitor SP600125 promotes chondrocyte proliferation but inhibits ECM synthesis. Here, we aimed to construct a three-dimensional cartilage regeneration model using a polyglycolic acid/polylactic acid scaffold in combination with chondrocytes to investigate the effect of different culture modes with CM and SP600125 on *in vitro* cartilage regeneration and their long-term outcomes *in vivo* systematically. Our results demonstrate that the long-term combination of CM and SP600125 made up for each other and maximized their respective advantages to obtain optimal cartilage regeneration *in vitro*. Moreover, the long-term combination achieved stable cartilage regeneration after implantation *in vivo* with a relatively low initial cell-seeding concentration. Therefore, the long-term combination of CM and SP600125 enhanced *in vitro* and *in vivo* cartilage regeneration stability with fewer initial seeding cells and thus optimized the cartilage regeneration culture system.

## Graphical Abstract

**Figure:**
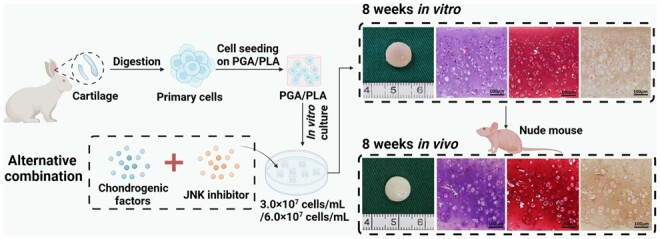

## Introduction

Cartilage defects are a common refractory disease [1, 2]. Fortunately, tissue engineering technology has made it possible to address this challenge [3, 4]. The core principle of cartilage tissue engineering is to establish a cell-scaffold construct and implant it into the defect area, where it can regenerate functional tissues similar to native tissue [5, 6]. The key to successful engineered cartilage regeneration and stability after implantation into the *in vivo* environment is to retain sufficient active cells with physiological functions, generate abundant cartilage extracellular matrix (ECM) deposition and maintain a low level of cartilage ECM degradation. To construct such an efficient engineered cartilage regeneration culture system, it is insufficient to rely solely on the construction of cell-scaffold composites; some effective exogenous factors must also be introduced.

Chondrocytes are the most commonly used cells for cartilage tissue engineering, but they are inevitably associated with limited cell supplies [7, 8] and are prone to lose their phenotype (dedifferentiate) [9]. Moreover, the current chondrogenic medium (CM) effectively promotes cartilage ECM production and leads to the redifferentiation of dedifferentiated chondrocytes; however, it negatively affects chondrocyte survival [10], leading to the regenerated cartilage failing to maintain a high content of active cells after implantation *in vivo* and eventually achieving poor cartilage regeneration. Strategies targeting the c-Jun N-terminal kinase (JNK) pathway remarkably reduce chondrocyte apoptosis in osteoarthritis (OA) [11–13]. Our previous studies have confirmed that the specific JNK inhibitor SP600125 can promote chondrocyte proliferation at the cellular and tissue levels *in vitro* and that the combined application of CM enhanced the synthesis and deposition of cartilage ECM [10]. This may be a feasible way to achieve satisfactory cartilage regeneration with a relatively low initial cell-seeding concentration and overcome the lack of active cells after implantation to improve cartilage regeneration quality *in vitro* and *in vivo*.

During multicellular organism development, cells proliferate before commencing functional differentiation [14]. During the differentiation of primordial cells into specific differentiated cells, their proliferation ability is limited [15, 16]. As cells differentiate and acquire specific physiological functions, they may enter the G0 phase and then lose their ability to divide, stopping cell proliferation [17, 18]. Our previous study demonstrated that SP600125 significantly promoted chondrocyte proliferation while inhibiting ECM secretion and cartilage differentiation. The current chondrogenic culture system promotes cartilage ECM production but impairs chondrocyte survival [10]. Since cell proliferation and differentiation belong to two different cell cycles and stages, whether phased application of different acting factors (i.e. first applying SP600125 to promote chondrocyte proliferation in the early stage and then replacing it with CM to promote chondrocyte differentiation and cartilage ECM synthesis) will achieve satisfactory cartilage regeneration is still unknown. Several issues must be clarified to determine the optimal culture mode of CM and SP600125 for cartilage regeneration. First, will phase application of CM and SP600125 enhance *in vitro* cartilage regeneration? Second, what is the outcome of regenerated cartilage treated with SP600125 *in vitro* after *in vivo* implantation? Third, can SP600125 enhance cartilage regeneration *in vitro* and *in vivo*, with fewer initial seeding cells? Therefore, we aimed to establish a three-dimensional cartilage regeneration model with polyglycolic acid/polylactic acid (PGA/PLA) scaffolds in combination with chondrocytes to investigate the effect of different culture modes with CM and SP600125 on *in vivo* cartilage regeneration systematically.

## Materials and methods

### Group design

For the *in vitro* cartilage regeneration model, high (6.0 × 10^7^ cells/ml) and low (3.0 × 10^7^ cells/ml) concentrations of cells were seeded onto the scaffolds. According to the period of application of 0.02 mg/ml SP600125 (a specific JNK pathway inhibitor, abbreviated as SP in grouping labels; AdooQ Bioscience, Irvine, CA) and CM (Dulbecco's modified eagle medium [DMEM] containing 10 ng/ml TGFβ1 [R&D Systems, Minneapolis, MN], 40 ng/ml dexamethasone [Sigma-Aldrich, St. Louis, MO], 100 ng/ml IGF-1 [R&D Systems] and other supplements; serum-free [7]), all samples were divided into six groups and cultured *in vivo* for 8 weeks. Chondrocytes from these six groups were obtained from the same rabbit. The overall group design of *in vitro* study is shown in Table 1.

**Table 1.** Group design of *in vitro* culture modes

Cell-seeding concentration (cells/ml)	First 10 days			Next 46 days			Label
	RM	CM	SP600125	RM	CM	SP600125	
H: 6.0 × 10^7^	+	−	−	+	−	−	HRM
L: 3.0 × 10^7^	+	−	−	+	−	−	LRM
	+	−	+	+	−	−	LRM + SP-RM
	+	−	−	−	+	−	LRM-CM
	+	−	+	−	+	−	LRM + SP-CM
	−	+	+	−	+	+	LCM + SP

H, high concentration; L, low concentration; SP, SP600125.

To study the *in vivo* outcomes of *in vitro* cultured regenerated cartilage treated with SP600125, the optimal group of the *in vitro* study and simple CM groups with high and low cell-seeding concentrations were cultured *in vitro* for 8 weeks and then implanted into nude mice subcutaneously. The overall group design of the *in vivo* study is shown in Table 2.

**Table 2.** Group design of *in vivo* outcomes

Cell-seeding concentration (cells/ml)	RM	CM	SP600125	Label
H: 6.0 × 10^7^	−	+	−	HCM
L: 3.0 × 10^7^	−	+	−	LCM
	−	+	+	LCM + SP

H, high concentration; L, low concentration; SP, SP600125.

### Isolation and culture of auricular chondrocytes

Rabbit auricular chondrocytes were harvested as previously described [19]. Chondrocytes from passage two were collected and used. The Ethical Committee of Shanghai Ninth People's Hospital affiliated with Shanghai Jiao Tong University School of Medicine approved all animal study protocols.

### Preparation of cell-scaffold constructs

PGA fibers (20 mg; National Tissue Engineering Center of China, Shanghai, China) were prepared to form cylinders by molds with a diameter of 9 mm and a thickness of 2 mm. Then, 1% PLA (Sigma, St. Louis, MO) solution was added dropwise to solidify the shape of the scaffolds [20]. After sterilization with a 75% ethanol solution for 30 min, the scaffolds were washed three times with phosphate-buffered saline for the following experiments. Harvested chondrocytes with different concentrations of 6.0 × 10^7^ cells/ml and 3.0 × 10^7^ in a regular medium (RM; DMEM containing 10% fetal bovine serum) were seeded onto each scaffold, followed by a 4-h pre-incubation [21]. Subsequently, different culture media were added according to group design (Tables 1 and 2).

### Biocapacity of the scaffolds

#### Scanning electron microscopy (SEM)

The microstructure of the PGA/PLA scaffolds and ECM deposition on the surfaces of scaffolds after culturing *in vitro* for 12 h, 4 days and 7 days were examined using SEM (Philips XL-30, Amsterdam, Netherlands). After being fixed overnight in 2.5% glutaraldehyde at 4°C, all samples were dehydrated in a graded series of ethanol solutions and then examined using SEM [22].

#### Cell-seeding efficiency

Chondrocytes with a concentration of 3.0 × 10^7^ cells/ml were seeded onto PGA/PLA scaffolds and cultured in different culture systems for 24 h. The samples were transferred for subsequent experiments and the remaining cells in the culture dishes were collected and counted. The cell-seeding efficiencies of scaffolds in different culture systems were calculated based on the formula [23]:


(total cell number - remaining cell number)/total cell number×100%

#### Cell proliferation evaluation

After 4 h, 1, 4 and 7 days of culture, the DNA content of samples in different culture systems was assessed using the DNA quantification assay (PicoGreen dsDNA assay; Invitrogen, Carlsbad, CA), as described previously [24], to evaluate the proliferation capacity of chondrocytes on scaffolds.

#### Cell viability evaluation

After 1, 4 and 7 days of culture, the viability of chondrocytes on scaffolds in different culture systems was assessed using the live and dead cell viability assay (Invitrogen, Carlsbad, CA), and the results were examined using a confocal microscope (Nikon, Japan) [25].

### Subcutaneous implantation in nude mice

Male nude mice aged 6–8 weeks were used for the *in vivo* study. After anesthetization with a small animal gas anesthetic and disinfection with 75% alcohol, their back skins were cut open, and the skin and muscle tissues were separated to the left, right and upper, respectively. Three groups of regenerated cartilage were placed under the skins of nude mice to ensure separation. The skin was intermittently sutured with 5–0 suture, and the wound was disinfected with 75% alcohol [26]. After 8 weeks, the nude mice were sacrificed, and samples from the three groups were taken for subsequent evaluation (Supplementary Fig. S1).

### Histological and immunohistochemical evaluations

Tissue samples were fixed in 4% paraformaldehyde for 48 h, embedded in paraffin and sectioned into 5-μm sections. The tissue sections were then stained with hematoxylin and eosin, safranin O and type II collagen (mouse anti-human type II collagen monoclonal antibody, 1:100; Santa Cruz Biotechnology, Dallas, TX), as previously described [27–29].

### Biomechanical and biochemical evaluations

After *in vitro* and *in vivo* culture, all samples were harvested and weighed. The volume was measured using the water displacement method [30]. The biomechanical properties of the samples in the different groups were measured using a biomechanical analyser (Instron-5542, Canton, MA), as previously described. Young’s modulus was calculated based on the stress-strain curves [31].

After mechanical analysis, tissue samples were minced for quantification of total collagen, glycosaminoglycan (GAG) and DNA using the hydroxyproline assay, the dimethyl methylene blue assay method and the PicoGreen dsDNA assay, respectively, as described previously [32–34].

### Quantitative real-time polymerase chain reaction analysis

Total RNA was extracted and reverse transcribed as previously described. The quantitative real-time polymerase chain reaction (qRT-PCR) was performed following the manufacturer’s protocol (Thermo Fisher Scientific, Waltham, MA). Expression levels of chondrogenesis-related genes (*ACAN*, *COLII A1* and *Sox9*) and catabolism-related genes (*TNF-α*, *IL-1β* and *MMP13*) were analyzed. Results were analyzed using the 2^-ΔΔ^CT method and normalized to the endogenous reference gene β-actin [35, 36]. The primer sequences are listed in Supplementary Table S1.

### Statistical analysis

Quantitative data were collected from at least three replicates and were presented as mean ± standard deviation. Statistical significance was analyzed using a one-way analysis of variance followed by post hoc tests with the Student–Newman–Keuls method using IBM SPSS Statistics (v.25; IBM Corp., Armonk, NY). Statistical significance was set at *P* < 0.05.

## Results

### Biocompatibility of cell-scaffold constructs

**Figure 1.**
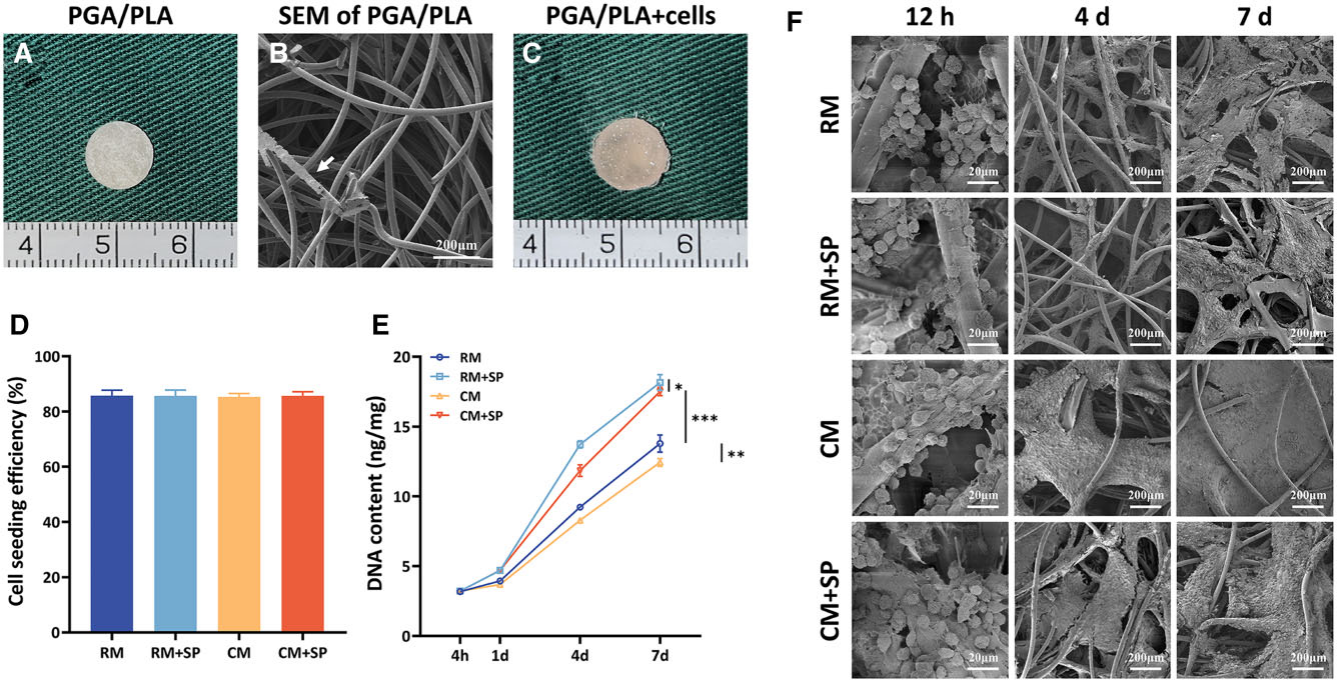
Preparation of *in vitro* cell-scaffold constructs. Gross observation (**A**), SEM (**B**, the arrow shows the PLA coating) of scaffolds. Immediate gross view of the cell-scaffold construct after cell seeding (**C**), cell-seeding efficiency (D) and DNA contents (E) of 4 h, 1 day, 4 days and 7 days after cell seeding. SEM images (**F**) of cell-scaffold construct in different culture systems after 12 h, 4 days and 7 days. SP, SP600125 (a specific JNK pathway inhibitor).

The PGA/PLA scaffold appeared as a porous cylindrical scaffold with a 9-mm diameter and 2-mm thickness (Fig. 1A). The SEM showed the ultrastructure of the interlaced PGA fiber and PLA coating (Fig. 1B). After seeding with 3.0 × 10^7^ cells/ml chondrocytes, the cell-scaffold constructs were cultured in different culture systems for biocompatibility evaluations (Fig. 1C). No significant difference was observed in the groups' cell-seeding efficiency and initial DNA content (Fig. 1D and E). With increasing culture time, the DNA content of all groups gradually increased. The groups treated with SP600125 showed an upward trend, suggesting more rapid cell proliferation. Meanwhile, the group treated with CM showed lower levels of DNA content, indicating a negative effect on cell proliferation. However, adding SP600125 weakened this adverse effect and achieved a higher DNA content in CM + SP than in RM. These results confirmed that SP600125 promoted cell proliferation and reversed CM’s inhibition of cell survival (Fig. 1E).

The SEM evaluated ECM deposition on the scaffolds’ surface (Fig. 1F). After 12 h of cell-seeding, the chondrocytes still appeared round in shape and adhered to the PGA fibers. After 4 days, the chondrocytes secreted ECM, and ECM deposition in the CM was more abundant. SP600125 inhibited ECM secretion in RM + SP, but adding CM significantly diminished this negative effect in CM + SP. After 7 d, more ECM was observed in all groups, and the fiber interspaces were covered in CM and CM + SP. SEM examination showed satisfactory biocompatibility of the scaffolds, and SP600125 reduced ECM synthesis, but adding CM reversed this.

**Figure 2.**
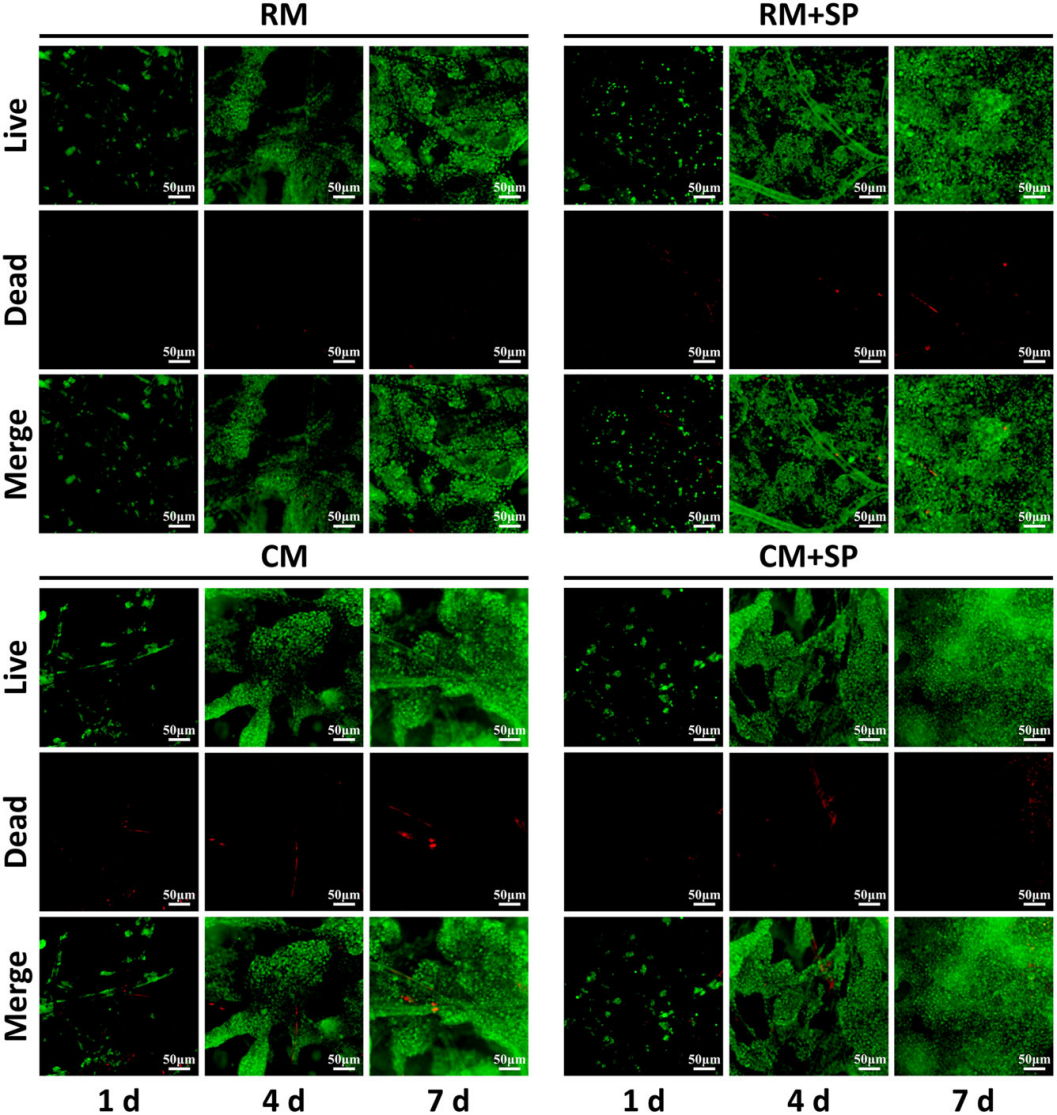
Cell viability of cells on scaffolds in different culture systems. Live/dead fluorescence staining revealed that the viable chondrocytes gradually proliferated with the increase in culture time in all groups. Adding SP600125 led to more significant cell proliferation in RM + SP and CM + SP groups. SP, SP600125 (a specific JNK pathway inhibitor).

Cell viability was evaluated using live/dead fluorescence staining (Fig. 2). The chondrocytes proliferated well with time in all groups, and few dead cells were observed, indicating the good biocompatibility of the scaffolds. More significant cell proliferation was observed in the RM + SP and CM + SP groups, indicating the cell proliferation-promoting effect of SP600125.

### Tissue-engineered cartilage regeneration *in vitro* with different culture modes

#### Gross and histological evaluation of in vitro cartilage regeneration

**Figure 3.**
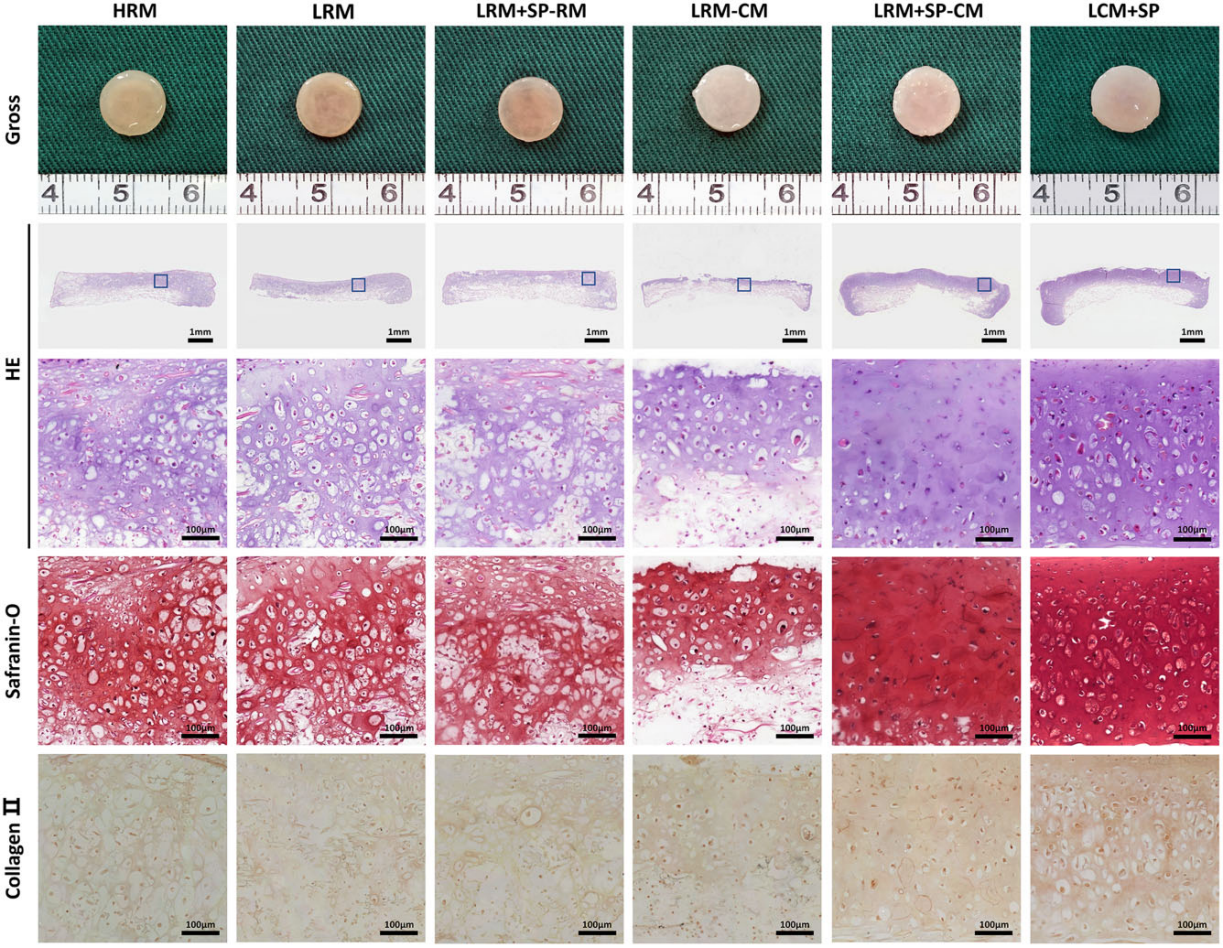
Gross view and histology of *in vitro* regenerated cartilages with different culture modes. Tissue samples showed different appearances and tissue structures. The regenerated tissue in groups treated with CM obtained a more mature cartilage-like appearance and structure. The LRM + SP-CM group with the phased application of SP600125 had an effect; however, the cartilage regeneration result was less mature than that of LCM + SP, obtaining the optimal cartilage regeneration *in vitro* with more mature cartilage lacunae and stronger staining for cartilage-specific ECM molecules. H, high cell-seeding density (6.0×10^7^ cells/ml); L, low cell-seeding density (3.0×10^7^ cells/ml); SP, SP600125 (a specific JNK pathway inhibitor).

The shape of the regenerated cartilage in each group remained relatively complete, and the scaffold materials on the surface had been fully degraded and replaced with new cartilage-like tissues. Groups treated with RM (HRM, LRM and LRM + SP-RM) had a light-yellow appearance and looser tissue texture. Groups treated with CM (LRM-CM, LRM + SP-CM and LCM + SP) formed a more mature cartilaginous tissue appearance. These results showed that the effects of different culture modes on the secretion and deposition of cartilage ECM led to general differences and that adding CM promoted cartilage regeneration (Fig. 3).

A histological examination was performed to study further the effects of each culture mode on the fine structure and composition of cartilage regeneration *in vitro.* After 8 weeks of culture *in vitro*, the scaffold materials of each group degraded completely. However, groups treated with CM formed more mature cartilaginous tissues, showing richer ECM deposition, more typical cartilage lacunae and stronger GAG and type II collagen staining. The tissue structures were disordered and loose in the RM group, indicating a relatively immature state of regenerated cartilage.

Since the initial cell-seeding concentration of HRM was higher than that of LRM, the histological results showed that HRM had more cells and more ECM secretion, probably because numerous active cells secreted cartilage-specific ECM. Compared to LRM, SP600125 was used to promote cell proliferation in the first 10 d in LRM + SP-RM. Histological results showed that LRM + SP-RM had more cells, but less ECM deposition than LRM. These results showed that the SP600125 inhibitory effect on cartilage ECM synthesis might persist after withdrawal. Moreover, chondrocytes with inhibited differentiation function still had function loss after returning to routine culture. Due to the lower cell content, LRM-CM formed a typical cartilage-like tissue, but the cartilage layer was thin. Only the sample’s surface formed cartilaginous tissue, and the lower layer comprised scaffold materials. In the LRM + SP-CM group, SP600125 was used to promote cell proliferation and increase active chondrocytes in the early stage, and CM was used to promote cell differentiation and cartilage ECM synthesis. Compared with LRM-CM, LRM + SP-CM had a higher cell content, richer secretion of cartilage ECM and stronger GAG and type II collagen expressions. However, LCM + SP had the most mature cartilaginous tissue formation and the strongest staining of GAG and type II collagen, accompanied by the highest active cells.

These results showed that the phased application of CM and SP600125 had an effect; however, the cartilage regeneration result was less mature than that of the long-term combined group, achieving optimal cartilage regeneration *in vitro*. This suggests that the concept that cell proliferation and differentiation belong to two different stages in our past cognition may be incomprehensive rather the stages are interrelated.

#### Quantitative determinations of in vitro cartilage regeneration

**Figure 4.**
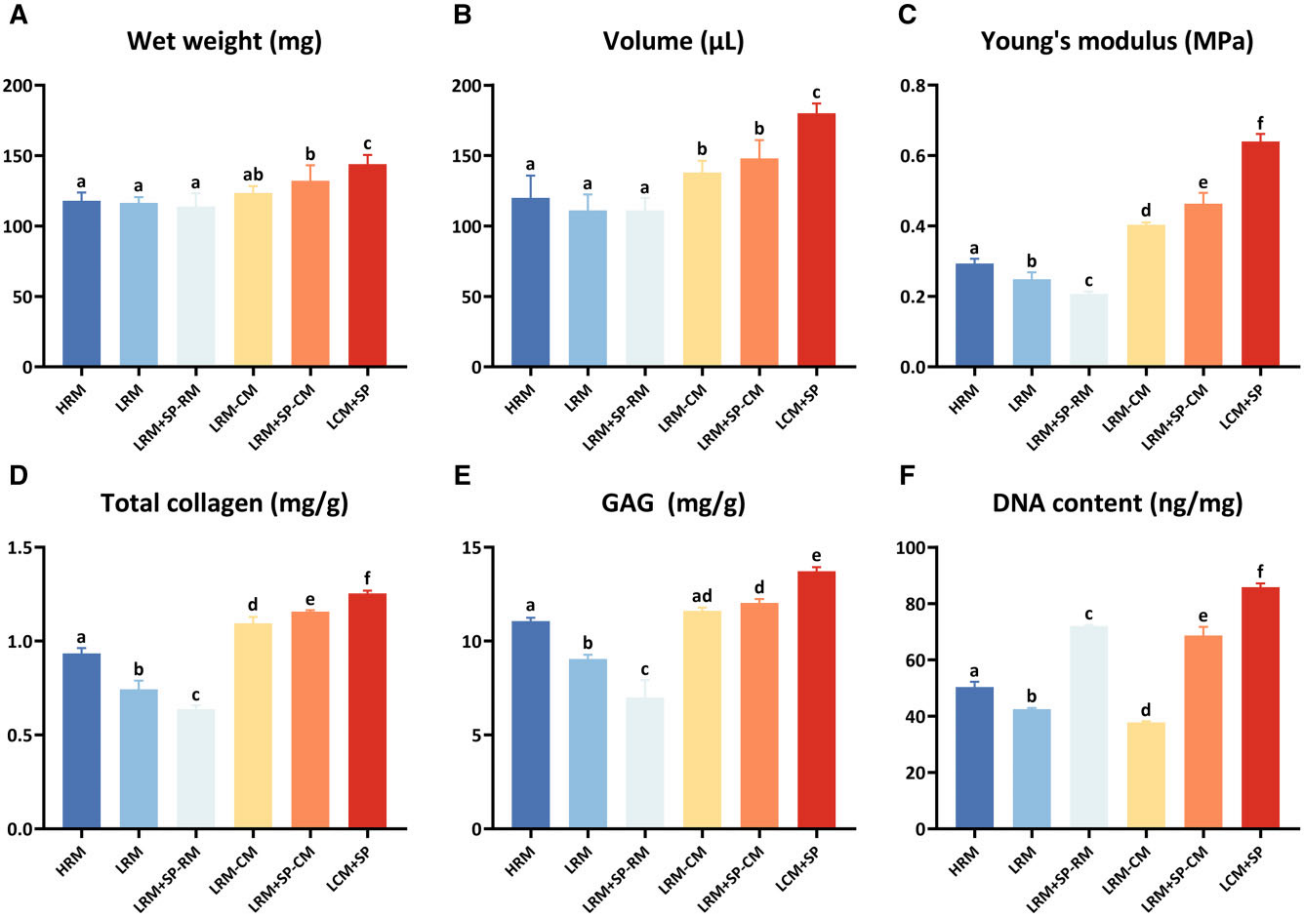
Quantitative evaluations of *in vitro* regenerated cartilages with different culture modes. Groups with CM had higher wet weight (**A**), volume (**B**), Young’s modulus (**C**), total collagen (**D**) and GAG contents (**E**) than the RM groups. The LCM + SP group had the optimal cartilage regeneration result *in vitro*. The DNA content (**F**) revealed that the cell number of groups treated with SP600125 was significantly higher. Owing to the inhibition of cell survival by CM, the LRM-CM group obtained the lowest DNA content; due to the chondrocyte proliferation-promoting effect of SP600125, the LCM + SP group obtained the highest DNA content. H, high cell-seeding density (6.0 × 10^7^ cells/ml); L, low cell-seeding density (3.0 × 10^7^ cells/ml); SP, SP600125 (a specific JNK pathway inhibitor).

Quantitative determinations of samples in each group were performed to verify further the histological results. CM groups had higher wet weight, volume, biomechanical strength and total collagen and GAG contents. These results demonstrated that CM promoted cartilage ECM secretion and the differentiation and maturation of regenerated cartilage *in vitro*. The LCM + SP group exhibited optimal cartilage regeneration *in vitro* (Fig. 4A–E).

There was no significant difference in wet weight and volume among the RM groups; however, the CM groups had significantly higher wet weight and volume than the RM groups. LRM + SP-CM had higher wet weight and volume than LRM-CM; however, they were lower than LCM + SP (Fig. 4A and B). There was a significant difference among RM groups in mechanical properties and related quantification of cartilage ECM components (total collagen and GAG quantification). HRM with the highest cell content showed the highest mechanical strength, total collagen and GAG contents. Owing to the continuation of SP600125 inhibitory effect on ECM synthesis, LRM + SP-RM showed the lowest in these directions. CM groups showed higher levels of biochemical and biomechanical parameters. Consistent with the histological results, LCM + SP with a long-term combination of CM and SP600125 resulted in optimal *in vitro* cartilage regeneration (Fig. 4C–E).

The results showed that groups treated with SP600125 had higher DNA content than other groups, consistent with the results of the JNK inhibitor promoting chondrocyte proliferation in a previous study. Similarly, due to CM’s negative effect on cell survival, LRM-CM had the lowest DNA content. The JNK inhibitor effectively promoted cell proliferation during long-term treatment in LCM + SP, leading to the highest DNA content. This also maintains long-term stability after implantation *in vivo* (Fig. 4F).

These results showed that LCM + SP with a long-term combination of SP600125 and CM resulted in optimal cartilage regeneration *in vitro*.

#### Characteristic gene expression of in vitro cartilage regeneration

**Figure 5.**
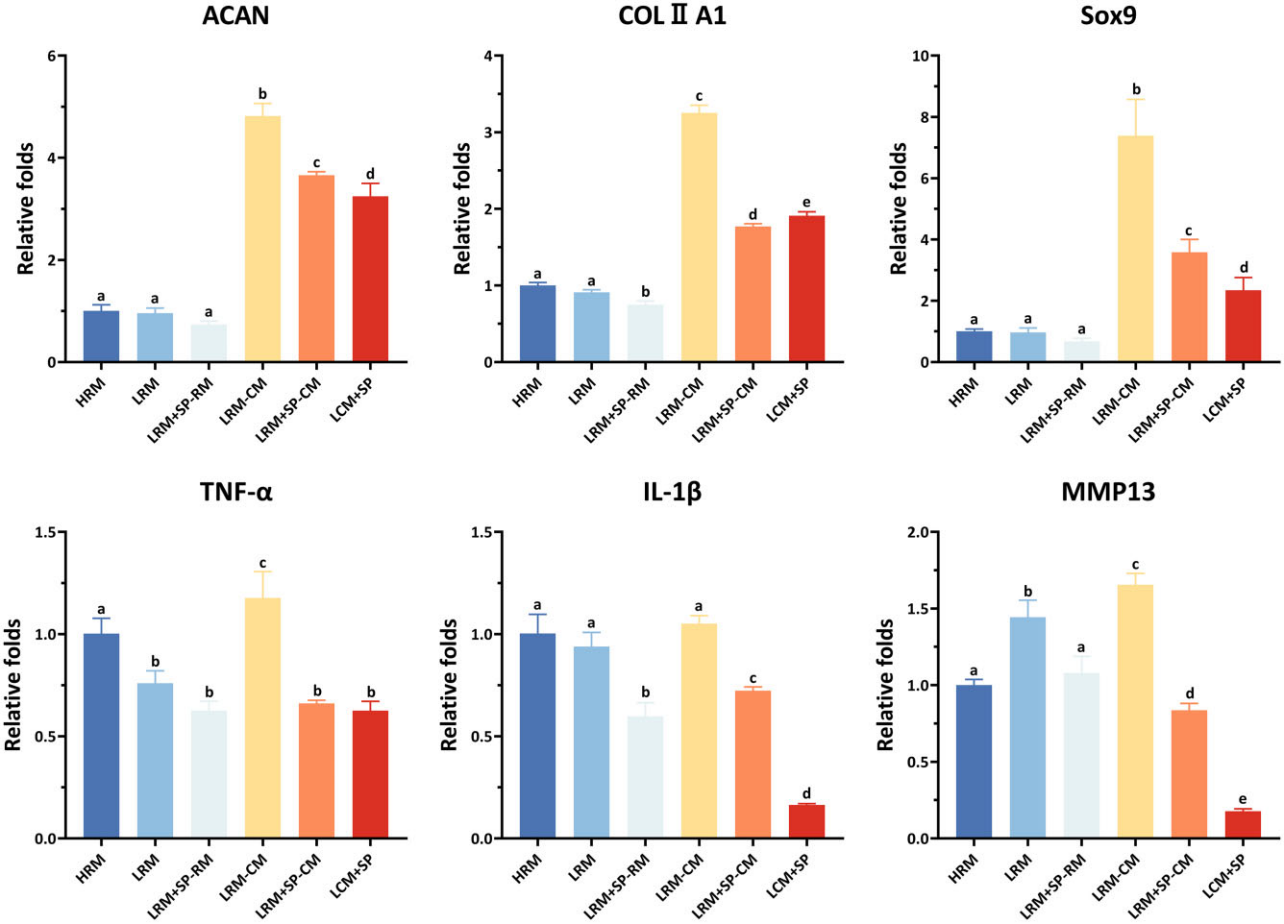
Analysis of the chondrogenesis-related and catabolism-related genes of *in vitro* regenerated cartilages with different culture modes. Expression of chondrogenesis genes (*ACAN*, *COLII A1* and *Sox9*) was downregulated in groups treated with SP600125, and adding CM diminished the negative effect. Expression of catabolism-related genes (*TNF-α*, *IL-1β* and *MMP13*) was also decreased in groups treated with SP600125, reducing the inflammatory response and cartilage ECM degradation. Chondrogenesis genes in the LCM + SP group were downregulated; however, they were higher than that in RM groups, and the inflammatory and degradation-related genes in the LCM + SP group were the lowest. H, high cell-seeding density (6.0×10^7^ cells/ml); L, low cell-seeding density (3.0×10^7^ cells/ml); SP, SP600125 (a specific JNK pathway inhibitor).

Chondrogenesis-related and catabolism-related genes were examined by qRT-PCR (Fig. 5). The expression levels of chondrogenesis-related genes (*ACAN*, *COLII A1* and *Sox9*), were downregulated in groups treated with SP600125. However, adding CM weakened this adverse effect. In addition, *TNF-α*, *IL-1β* and *MMP13* expressions were also downregulated, indicating reduced inflammatory response and ECM degradation. LCM + SP had lower levels of chondrogenesis-related genes than LRM-CM due to the inhibition of ECM synthesis by SP600125. However, LCM + SP also had the lowest cartilage catabolism-related genes (*TNF-α*, *IL-1β* and *MMP13*), whereas LRM-CM had the highest expression. LCM + SP has the greatest potential for cartilage regeneration *in vitro* because good cartilage regeneration depends on synthesis and degradation balance.

### *In vivo* outcomes of tissue-engineered cartilage regeneration

#### Gross view and histology of in vitro and in vivo cartilage regeneration

This study’s results showed that LRM + SP-CM with the phased application of CM and SP600125 had better cartilage regeneration; however, it was inferior to LCM + SP with a long-term combination. Nevertheless, the combined application of CM and SP600125 resulted in optimal *in vitro* cartilage regeneration. Therefore, LCM + SP was selected as the experimental group, and groups cultured in simple CM with high and low cell-seeding concentrations were used as the control groups to evaluate their cartilage regeneration effects *in vitro* and their outcomes *in vivo*.

**Figure 6.**
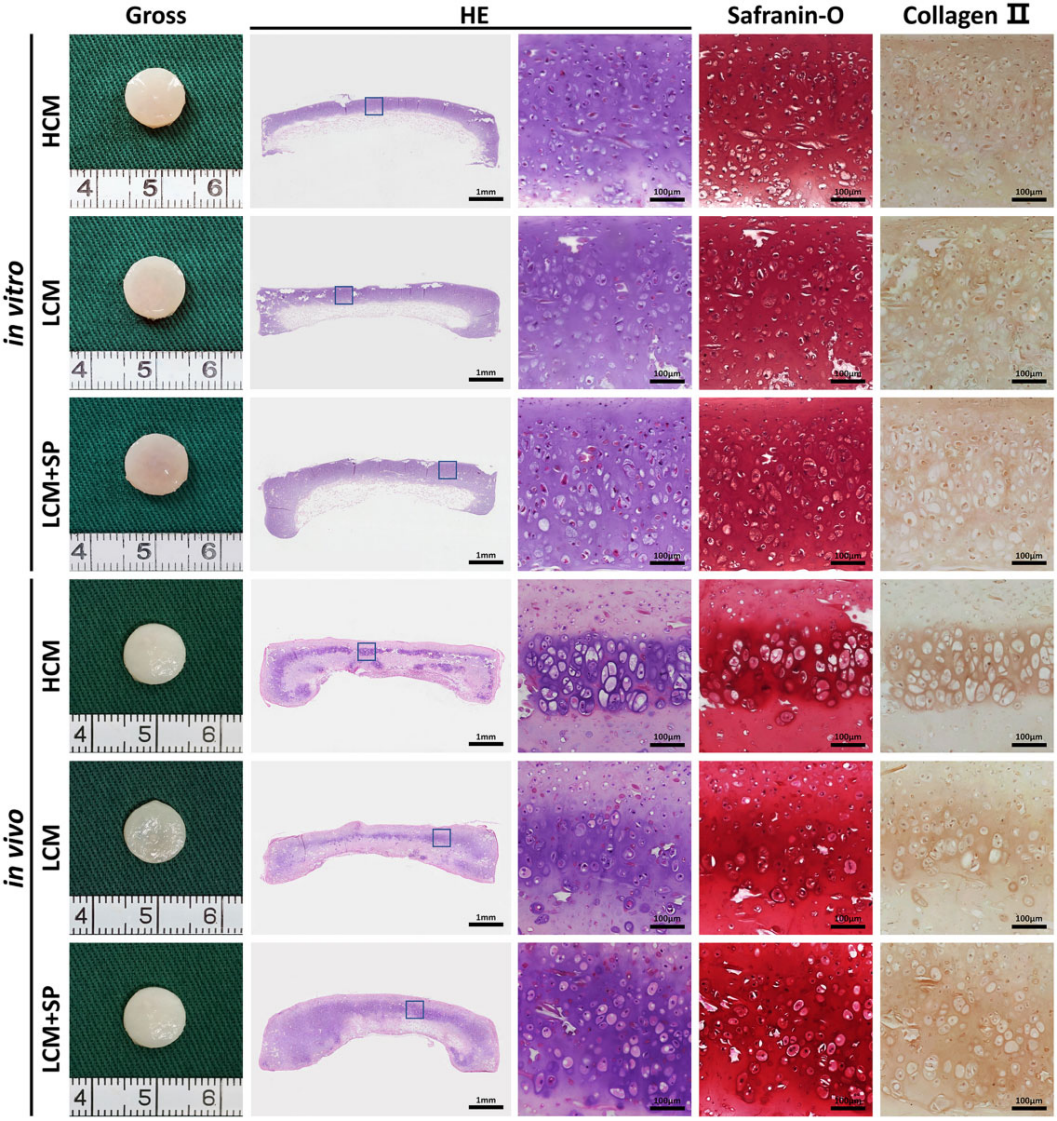
Gross view and histology of *in vitro* and *in vivo* regenerated cartilages cartilage-like tissues were formed in all three groups *in vitro*. After 8 weeks of *in vivo* implantation, cartilage formation matured with time. However, the formation of cartilage tissue was more uniform in the LCM + SP group, while only cartilage islands were distributed in HCM and LCM groups. H, high cell seeding density (6.0×10^7^ cells/ml); L, low cell seeding density (3.0×10^7^ cells/ml); SP, SP600125 (a specific JNK pathway inhibitor).

After 8 weeks of *in vitro* culture, smooth and ivory-white cartilaginous tissues were formed in all groups. Histological examination showed that mature cartilage lacunae formed in all groups. The expression of GAG and type II collagen, specific components of cartilage ECM, were positive. Regarding cell content, HCM and LCM + SP had more nuclei stained blue-black than LCM. After 8 weeks of implantation *in vivo*, the cartilage in all groups appeared similar to natural cartilage, and the shapes were well maintained. Histological examination revealed that the cartilage lacuna structures of all three groups were more mature than those *in vitro*, and the expression of GAG and type II collagen was positive. Cartilage tissue formation was uniform and homogeneous in the LCM + SP group, while only cartilage islands were distributed in the HCM and LCM groups. These results showed that cartilage formation matured over time after transplantation *in vivo*. LCM + SP achieved more homogeneous and stable cartilage regeneration *in vivo*, possibly due to the retention of sufficient active cells and a lower degradation activity (Fig. 6).

#### Quantitative determinations of in vitro and in vivo cartilage regeneration

**Figure 7.**
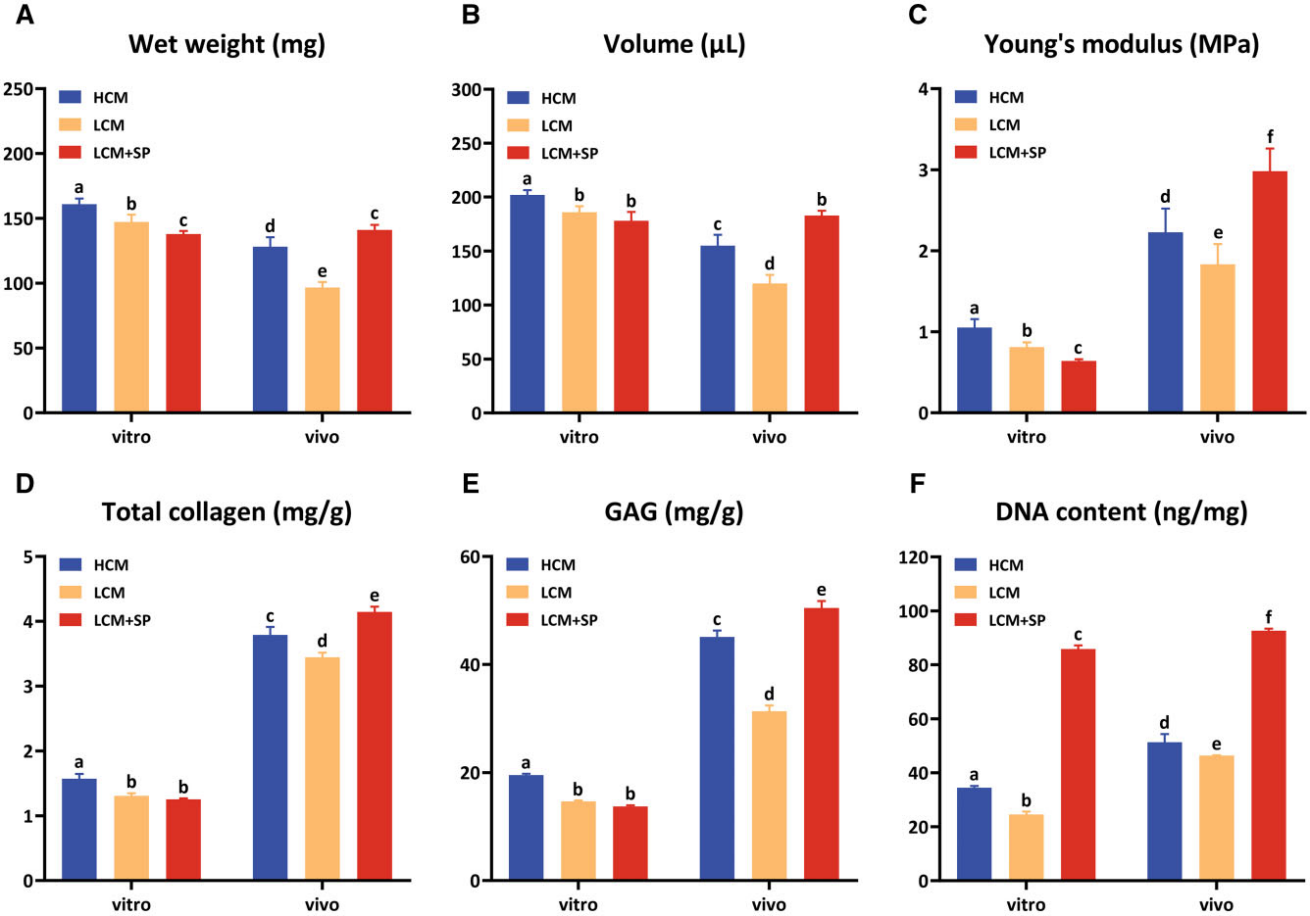
Quantitative evaluations of *in vitro* and *in vivo* regenerated cartilages. In the *in vitro* culture stage, the LCM + SP group obtained lower levels of wet weight (**A**), volume (**B**), mechanical strength (**C**), total collagen (**D**) and GAG contents (**E**) but the highest DNA content. After 8 weeks of implantation *in vivo*, the outcomes reversed and the LCM + SP group obtained the highest level of all cartilage regeneration-related evaluations, indicating that the content of active cells and degradation activity were the main determinants of cartilage regeneration *in vivo*. H, high cell seeding density (6.0×10^7^ cells/ml); L, low cell seeding density (3.0×10^7^ cells/ml); SP: SP600125 (a specific JNK pathway inhibitor).

Quantitative determinations further confirmed the histological results. In the *in vitro* culture stage, LCM + SP had a lower wet weight, volume, mechanical strength, total collagen and GAG contents than HCM, and there was no significant difference compared to LCM, except for wet weight (Fig. 7A–E). However, the DNA content of the LCM + SP group was the highest due to the promotion effect of SP600125 on chondrocyte proliferation (Fig. 7F). These results were consistent with those of a previous study.

After 8 weeks of implantation *in vivo*, the wet weight and volume of HCM and LCM decreased, whereas those of LCM + SP were well maintained (Fig. 7A and B). These results showed that in the enzyme-active environment *in vivo*, numerous active cells were retained and further performed their biological functions due to the inhibition of cartilage degradation and promotion of chondrocyte proliferation by SP600125, resulting in the good maintenance of the shape and size of *in vitro* regenerated cartilage. The mechanical properties and ECM component contents were reversed after 8 weeks of *in vivo* implantation. LCM + SP *in vitro* with the lowest mechanical strength and total collagen and GAG contents obtained the highest level after *in vivo* implantation (Fig. 7C–E). The DNA content increased in all groups after implantation, but the LCM + SP group had the highest active cells, the key reason for the reversal *in vivo* (Fig. 7F).

These results showed that the cartilage regeneration of LCM + SP was inferior to that of HCM and LCM *in vitro*; however, it was reversed after 8 weeks of implantation due to its numerous active cells and reduced degradation of cartilage ECM.

#### Characteristic gene expression of in vitro and in vivo cartilage regeneration

**Figure 8.**
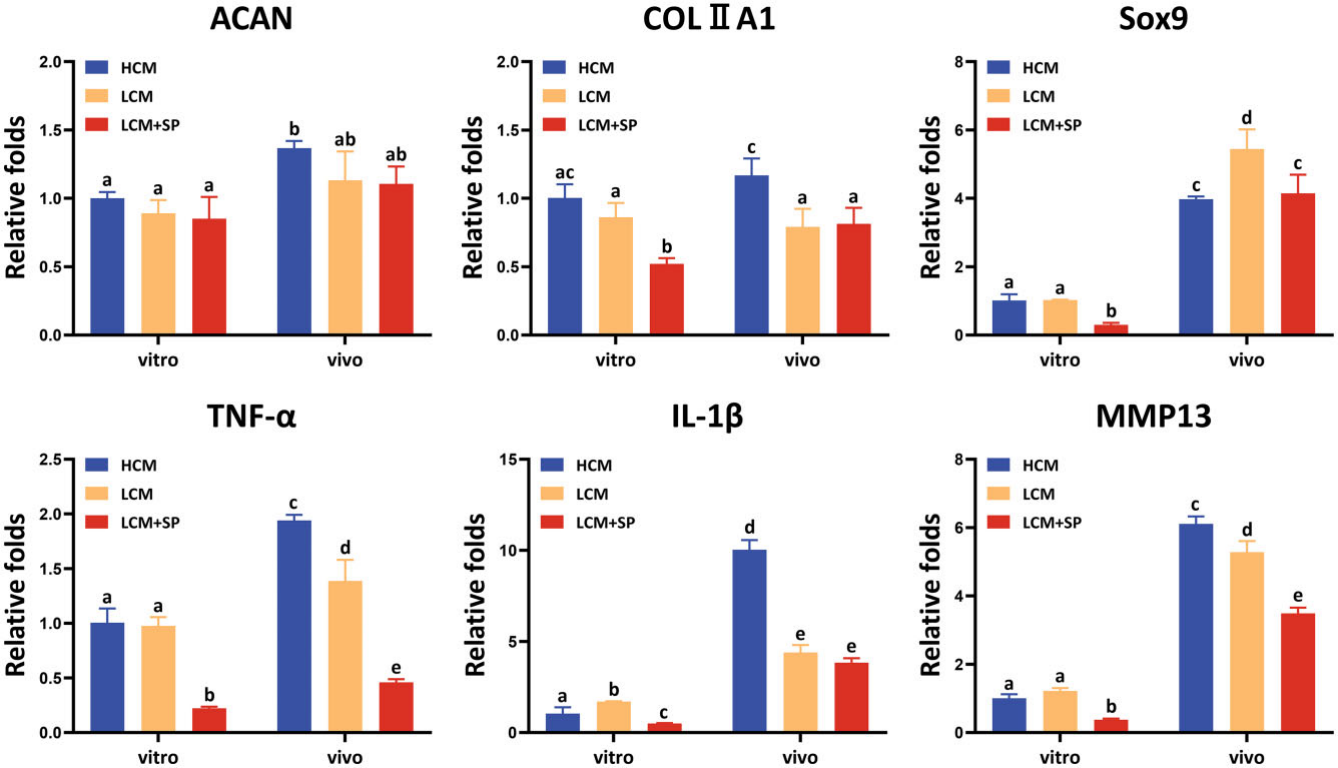
Analysis of chondrogenesis-related genes and catabolism-related genes of *in vitro* and *in vivo* regenerated cartilages. After 8 weeks of *in vivo* implantation, the expression levels of chondrogenesis and catabolism-related genes in all three groups were upregulated, indicating the tissue-engineered cartilage constructed *in vitro* further matured after implantation *in vivo* and regenerated cartilages had higher degradation activity in the *in vivo* environment. The LCM + SP group showed lower levels of chondrogenesis-related genes and lower catabolism-related genes both *in vitro* and *in vivo*. H, high cell seeding density (6.0×10^7^ cells/ml); L, low cell seeding density (3.0×10^7^ cells/ml); SP, SP600125 (a specific JNK pathway inhibitor).

Chondrogenesis-related and catabolism-related genes were analyzed using qRT-PCR (Fig. 8). Chondrogenesis-related genes were upregulated after implantation *in vivo* in all three groups, indicating that the *in vivo* environment is conducive to further maturation of regenerated cartilage. Expression of chondrogenesis-related genes of LCM + SP was relatively lower than those of HCM and LCM *in vitro* and *in vivo*. In contrast, the levels of LCM + SP degradation-related genes, including *TNF-α*, *IL-1β* and *MMP13,* remained the lowest. Importantly, in the complex *in vivo* environment, due to the influence of body fluids, degradation enzymes and other inflammatory factors, the degradation level of regenerated cartilage was upregulated, and a new balance between cartilage synthesis and degradation needs to be established. Thus, lower levels of cartilage degradation are important for *in vivo* cartilage regeneration. The lower expression of degradation-related genes was critical to maintaining the stability of regenerated cartilage *in vivo* when the expression of chondrogenesis-related genes is not low. Therefore, the LCM + SP combination exhibited the strongest cartilage regeneration potential *in vivo*.

## Discussion

Cartilage regeneration engineering is gaining increased attention owing to the promising results in cartilage injury repair. Moreover, developing an efficient engineered cartilage regeneration culture system and maintaining the stability of the regenerated cartilage after *in vivo* implantation remains challenging. Here, we combined the currently used CM and SP60015, a specific JNK inhibitor, to construct a new *in vitro-engineered* cartilage culture system. Our results demonstrated that the long-term combination of CM and SP600125 enhanced cartilage regeneration *in vitro* and *in vivo* with fewer initial seeding cells by increasing the number of active cells, improving ECM synthesis and reducing ECM degradation.

Chondrocytes are the only cells that exist in cartilage tissues [37]. Hence, satisfactory cartilage regeneration lies in the active cell quantities. However, strategies using chondrocytes as a cell source for cartilage tissue engineering are associated with limited cell supplies [7, 38]. Moreover, the CM currently used negatively affects cell survival, hindering the long-term stability of regenerated cartilage. Inhibiting the JNK pathway decreases chondrocyte loss in OA [13]. Our study confirmed the significant effect of the specific JNK inhibitor SP600125 on promoting chondrocyte proliferation. This may promote satisfactory cartilage regeneration with fewer initial cell seeding, reducing the demand for cartilage donors. Meanwhile, the constructed tissue-engineered cartilage with high cell content can be maintained after implantation *in vivo*.

Another decisive factor affecting the quality of cartilage regeneration is the metabolic balance between cartilage ECM synthesis and degradation [39, 40]. Satisfactory cartilage regeneration depends on sufficient ECM secretion and low-level degradation activity. Consistent with our previous report, our results demonstrated that SP600125 negatively affects cartilage ECM synthesis, as indicated by the inferiority of cartilage-related quantitative evaluations and the downregulation of chondrogenesis-related gene expression. Fortunately, the combination with CM reversed these negative effects and enhanced cartilage ECM production. Simultaneously, SP600125 decreased cartilage degradation, as evidenced by the downregulation of inflammation and degradation-related gene expression. Overall, the combination of CM and SP600125 compensated for each other and achieved abundant cartilage ECM production and low-level cartilage degradation, leading to satisfactory cartilage regeneration.

Cells proliferate for a certain period before commencing functional differentiation. In the process of cell differentiation, meaning that cells acquire specific physiological functions; their proliferation ability will gradually weaken and the ability of cell division is lost, stopping cell proliferation [17]. Since cell proliferation and differentiation belong to two different stages, whether the phased application of CM and SP600125, that is, promoting cell proliferation with SP600125 in the early stage and promoting cell differentiation with CM in the later stage, will obtain optimal cartilage regeneration is still unknown. Our results showed that the phased application of CM and SP600125 avoided the negative effects on cartilage regeneration; however, it was inferior to the long-term combination group in the number of active cells and the quality of regenerated cartilage. These results suggest that our previous understanding of cell proliferation and differentiation stages may be insufficient. Cell proliferation and differentiation may penetrate and affect each other in tissue formation. Qiu et al. discovered that preadipocytes could differentiate into adipocytes without DNA synthesis and mitotic clonal expansion [41], while Parker reported that activated B-lymphocytes simultaneously undergo numerous clonal expansions when they mature [42]. The evidence showed that although cell proliferation and differentiation are interrelated, they are independent of each other: cell proliferation is not accompanied by stagnation of differentiation and cell differentiation does not depend on cells exiting the proliferation cycle. Therefore, the long-term combination of CM and SP600125 can maximize their advantages and compensate for each other. Additionally, it can promote chondrocyte proliferation and differentiation, retain numerous active cells and secrete abundant specific cartilage ECM to achieve the optimal effect of cartilage regeneration.

*In vivo* cartilage regeneration involves a more complex physiological environment than *in vivo* regeneration [43, 44]. The shape, volume and long-term outcomes of regenerated cartilage constructed *in vitro* are affected by uncertain factors such as the pH of the body fluid, the tension of the surrounding tissues and degrading enzymes *in vivo*. Our results showed that, compared with the simple application of CM, the combination of CM and SP600125 did not have a significant advantage in the quality of cartilage regeneration in the *in vitro* culture stage, except for the high cell content. However, under the complex physiological and mechanical environment *in vivo*, it was unclear whether LCM + SP could have numerous active cells and better maintain the long-term stability of the regenerated cartilage. Our results demonstrated that the wet weight and volume of LCM + SP were maintained after 8 weeks of implantation *in vivo*. Similarly, the mechanical properties and content of cartilage-specific ECM components increased significantly and exceeded those of the simple CM groups. These results indicated that the induction effect of exogenous chondrogenic factors was lost in the complex *in vivo* environment. Additionally, further cartilage maturation depends more on cells with activity and normal physiological function [45]. SP600125 in *in vitro* culture promoted chondrocyte proliferation, resulting in the largest active cells after implantation *in vivo*, to reverse and obtain the optimal effect of cartilage regeneration *in vivo*. Chondrogenesis-related gene expression evaluations revealed that SP600125 inhibited ECM degradation. The degrading enzymes and inflammatory factors were low in the *in vitro* culture environment. However, there were numerous unstable factors in the *in vivo* environment; thus, inhibiting the degradation of cartilage ECM for cartilage regeneration is important. In general, regenerated cartilage cultured with the long-term combination of CM and SP600125 obtained sufficient active cells to secrete abundant cartilage-specific ECM and lower levels of degradation activity, to obtain more stable cartilage regeneration *in vivo*.

This study confirmed that the long-term combination of CM and SP600125 enhanced cartilage regeneration *in vitro* and *in vivo* with fewer initial seeding cells, thus optimizing the current cartilage regeneration culture system. However, downstream transcription factors of the JNK pathway are closely related to tumor occurrence and development [46, 47]. The dosage of the JNK inhibitor SP600125 used in this study was within safe doses; however, there was a lack of direct evidence to prove its biological safety in regulating cartilage regeneration. In future studies, we will comprehensively examine the biological safety of SP600125 and further determine the optimal application dose for regulating cartilage regeneration through karyotype analysis, gene mutation screening, proto-oncogene expression, tumorigenicity assay and other related aspects. In addition, a large animal articular cartilage defect model will be constructed as described previously in the future study [48], combined with a biodegradable scaffold loaded with SP600125 controlled-release microspheres, to further discuss the regulation effect of SP600125 on the regeneration of cartilage defects in large animals.

## Conclusion

In summary, the results of this study indicated that a long-term combination of CM and SP600125 obtained stable cartilage regeneration, both *in vitro* and *in vivo*, with fewer initial seeding cells. Therefore, few cartilage donors are required to achieve satisfactory cartilage regeneration, thus enhancing the efficiency and stability of the engineered cartilage regeneration system.

## Supplementary Material

rbad079_Supplementary_Data

## Data Availability

The data that supports the findings of this study are available within the article and its supplementary material.

## References

[1] Chen S , Fu P, Wu H, Pei M. Meniscus, articular cartilage and nucleus pulposus: a comparative review of cartilage-like tissues in anatomy, development and function. Cell Tissue Res 2017;370:53–70. 28413859 10.1007/s00441-017-2613-0 PMC5645221

[2] He T , Li B, Colombani T, Joshi-Navare K, Mehta S, Kisiday J, Bencherif SA, Bajpayee AG. Hyaluronic Acid-Based Shape-Memory cryogel scaffolds for focal cartilage defect repair. Tissue Eng Part A 2021;27:748–60. 33108972 10.1089/ten.TEA.2020.0264

[3] Yang J , Zhang YS, Yue K, Khademhosseini A. Cell-laden hydrogels for osteochondral and cartilage tissue engineering. Acta Biomater 2017;57:1–25. 28088667 10.1016/j.actbio.2017.01.036 PMC5545789

[4] Campos Y , Almirall A, Fuentes G, Bloem HL, Kaijzel EL, Cruz LJ. Tissue engineering: an alternative to repair cartilage. Tissue Eng Part B Rev 2019;25:357–73. 30913997 10.1089/ten.TEB.2018.0330

[5] Moreira-Teixeira LS , Georgi N, Leijten J, Wu L, Karperien M. Cartilage tissue engineering. Endocr Dev 2011;21:102–15. 21865759 10.1159/000328140

[6] Bakhshandeh B , Zarrintaj P, Oftadeh MO, Keramati F, Fouladiha H, Sohrabi-Jahromi S, Ziraksaz Z. Tissue engineering; strategies, tissues, and biomaterials. Biotechnol Genet Eng Rev 2017;33:144–72. 29385962 10.1080/02648725.2018.1430464

[7] He A , Liu L, Luo X, Liu Y, Liu Y, Liu F, Wang X, Zhang Z, Zhang W, Liu W, Cao Y, Zhou G. Repair of osteochondral defects with in vitro engineered cartilage based on autologous bone marrow stromal cells in a swine model. Sci Rep 2017;7:40489. 28084417 10.1038/srep40489 PMC5234019

[8] He A , Xia H, Xiao K, Wang T, Liu Y, Xue J, Li D, Tang S, Liu F, Wang X, Zhang W, Liu W, Cao Y, Zhou G. Cell yield, chondrogenic potential, and regenerated cartilage type of chondrocytes derived from ear, nasoseptal, and costal cartilage. J Tissue Eng Regen Med 2018;12:1123–32. 29139602 10.1002/term.2613

[9] He A , Ye A, Song N, Liu N, Zhou G, Liu Y, Ye X. Phenotypic redifferentiation of dedifferentiated microtia chondrocytes through a three-dimensional chondrogenic culture system. Am J Transl Res 2020;12:2903–15. 32655818 PMC7344067

[10] Zhang P , Liu Y, Jia L, Ci Z, Zhang W, Liu Y, Chen J, Cao Y, Zhou G. SP600125, a JNK-Specific inhibitor, regulates in vitro auricular cartilage regeneration by promoting cell proliferation and inhibiting extracellular matrix metabolism. Front Cell Dev Biol 2021;9:630678. 33816478 10.3389/fcell.2021.630678 PMC8010669

[11] Johnson GL , Nakamura K. The c-jun kinase/stress-activated pathway: regulation, function and role in human disease. Biochim Biophys Acta 2007;1773:1341–8. 17306896 10.1016/j.bbamcr.2006.12.009 PMC1995559

[12] Lu H , Wang W, Kang X, Lin Z, Pan J, Cheng S, Zhang J. Hydrogen (H2) alleviates osteoarthritis by inhibiting apoptosis and inflammation via the JNK signaling pathway. J Inflamm Res 2021;14:1387–402. 33880054 10.2147/JIR.S297622 PMC8053515

[13] Ge HX , Zou FM, Li Y, Liu AM, Tu M. JNK pathway in osteoarthritis: pathological and therapeutic aspects. J Recept Signal Transduct Res 2017;37:431–6. 28812968 10.1080/10799893.2017.1360353

[14] Wuelling M , Vortkamp A. Chondrocyte proliferation and differentiation. Endocr Dev 2011;21:1–11. 21865749 10.1159/000328081

[15] Zhu L , Skoultchi AI. Coordinating cell proliferation and differentiation. Curr Opin Genet Dev 2001;11:91–7. 11163157 10.1016/s0959-437x(00)00162-3

[16] Ruijtenberg S , van den Heuvel S. Coordinating cell proliferation and differentiation: antagonism between cell cycle regulators and cell type-specific gene expression. Cell Cycle 2016;15:196–212. 26825227 10.1080/15384101.2015.1120925 PMC4825819

[17] Brown G , Hughes PJ, Michell RH. Cell differentiation and proliferation–simultaneous but independent? Exp Cell Res 2003;291:282–8. 14644151 10.1016/s0014-4827(03)00393-8

[18] Dalton S. Linking the cell cycle to cell fate decisions. Trends Cell Biol 2015;25:592–600. 26410405 10.1016/j.tcb.2015.07.007 PMC4584407

[19] Li Y , Liu Y, Xun X, Zhang W, Xu Y, Gu D. Three-Dimensional porous scaffolds with biomimetic microarchitecture and bioactivity for cartilage tissue engineering. ACS Appl Mater Interfaces 2019;11:36359–70. 31509372 10.1021/acsami.9b12206

[20] Ding C , Qiao Z, Jiang W, Li H, Wei J, Zhou G, Dai K. Regeneration of a goat femoral head using a tissue-specific, biphasic scaffold fabricated with CAD/CAM technology. Biomaterials 2013;34:6706–16. 23773816 10.1016/j.biomaterials.2013.05.038

[21] Xue J , He A, Zhu Y, Liu Y, Li D, Yin Z, Zhang W, Liu W, Cao Y, Zhou G. Repair of articular cartilage defects with acellular cartilage sheets in a swine model. Biomed Mater 2018;13:025016. 29125133 10.1088/1748-605X/aa99a4

[22] Ci Z , Zhang Y, Wang Y, Wu G, Hou M, Zhang P, Jia L, Bai B, Cao Y, Liu Y, Zhou G. 3D cartilage regeneration with certain shape and mechanical strength based on engineered cartilage gel and decalcified bone matrix. Front Cell Dev Biol 2021;9:638115. 33718376 10.3389/fcell.2021.638115 PMC7952450

[23] Hou M , Tian B, Bai B, Ci Z, Liu Y, Zhang Y, Zhou G, Cao Y. Dominant role of in situ native cartilage niche for determining the cartilage type regenerated by BMSCs. Bioact Mater 2022;13:149–60. 35224298 10.1016/j.bioactmat.2021.11.007 PMC8843973

[24] Xu Y , Xu Y, Bi B, Hou M, Yao L, Du Q, He A, Liu Y, Miao C, Liang X, Jiang X, Zhou G, Cao Y. A moldable thermosensitive hydroxypropyl chitin hydrogel for 3D cartilage regeneration in vitro and in vivo. Acta Biomater 2020;108:87–96. 32268237 10.1016/j.actbio.2020.03.039

[25] Jia L , Zhang P, Ci Z, Zhang W, Liu Y, Jiang H, Zhou G. Immune-Inflammatory responses of an acellular cartilage matrix biomimetic scaffold in a xenotransplantation goat model for cartilage tissue engineering. Front Bioeng Biotechnol 2021;9:667161. 34150731 10.3389/fbioe.2021.667161 PMC8208476

[26] Li D , Zhu L, Liu Y, Yin Z, Liu Y, Liu F, He A, Feng S, Zhang Y, Zhang Z, Zhang W, Liu W, Cao Y, Zhou G. Stable subcutaneous cartilage regeneration of bone marrow stromal cells directed by chondrocyte sheet. Acta Biomater 2017;54:321–32. 28342879 10.1016/j.actbio.2017.03.031

[27] Jia L , Zhang Y, Yao L, Zhang P, Ci Z, Zhang W, Miao C, Liang X, He A, Liu Y, Tang S, Zhang R, Wang X, Cao Y, Zhou G. Regeneration of human-ear-shaped cartilage with acellular cartilage matrix-based biomimetic scaffolds. Appl Mater Today 2020;20:100639.

[28] Xia H , Zhao D, Zhu H, Hua Y, Xiao K, Xu Y, Liu Y, Chen W, Liu Y, Zhang W, Liu W, Tang S, Cao Y, Wang X, Chen HH, Zhou G. Lyophilized scaffolds fabricated from 3D-Printed photocurable natural hydrogel for cartilage regeneration. ACS Appl Mater Interfaces 2018;10:31704–15. 30157627 10.1021/acsami.8b10926

[29] Yin Z , Li D, Liu Y, Feng S, Yao L, Liang X, Miao C, Xu Y, Hou M, Zhang R, Zhang W, Liu W, Liu Y, Zhou G, Cao Y. Regeneration of elastic cartilage with accurate human-ear shape based on PCL strengthened biodegradable scaffold and expanded microtia chondrocytes. Appl Mater Today 2020;20:100724.

[30] Wang Y , Xu Y, Zhou G, Liu Y, Cao Y. Biological evaluation of acellular cartilaginous and dermal matrixes as tissue engineering scaffolds for cartilage regeneration. Front Cell Dev Biol 2020;8:624337. 33505975 10.3389/fcell.2020.624337 PMC7829663

[31] Chen W , Chen S, Morsi Y, El-Hamshary H, El-Newhy M, Fan C, Mo X. Superabsorbent 3D scaffold based on electrospun nanofibers for cartilage tissue engineering. ACS Appl Mater Interfaces 2016;8:24415–25. 27559926 10.1021/acsami.6b06825

[32] Xu Y , Guo Y, Li Y, Huo Y, She Y, Li H, Jia Z, Jiang G, Zhou G, You Z, Duan L. Biomimetic trachea regeneration using a modular ring strategy based on poly(sebacoyl diglyceride)/polycaprolactone for segmental trachea defect repair. Adv Funct Mater 2020;30:2004276.

[33] Chen W , Xu Y, Li Y, Jia L, Mo X, Jiang G, Zhou G. 3D printing electrospinning fiber-reinforced decellularized extracellular matrix for cartilage regeneration. Chem Eng J 2020;382:122986.

[34] Xu Y , Wang Z, Hua Y, Zhu X, Wang Y, Duan L, Zhu L, Jiang G, Xia H, She Y, Zhou G. Photocrosslinked natural hydrogel composed of hyaluronic acid and gelatin enhances cartilage regeneration of decellularized trachea matrix. Mater Sci Eng C Mater Biol Appl 2021;120:111628. 33545814 10.1016/j.msec.2020.111628

[35] Hao J , Bai B, Ci Z, Tang J, Hu G, Dai C, Yu M, Li M, Zhang W, Zhang Y, Ren W, Hua Y, Zhou G. Large-sized bone defect repair by combining a decalcified bone matrix framework and bone regeneration units based on photo-crosslinkable osteogenic microgels. Bioact Mater 2022;14:97–109. 35310359 10.1016/j.bioactmat.2021.12.013 PMC8892219

[36] Wu G , Lu L, Ci Z, Wang Y, Shi R, Zhou G, Li S. Three-Dimensional cartilage regeneration using engineered cartilage gel with a 3D-printed polycaprolactone framework. Front Bioeng Biotechnol 2022;10:871508. 35685090 10.3389/fbioe.2022.871508 PMC9171075

[37] Chinta ML , Velidandi A, Pabbathi NPP, Dahariya S, Parcha SR. Assessment of properties, applications and limitations of scaffolds based on cellulose and its derivatives for cartilage tissue engineering: a review. Int J Biol Macromol 2021;175:495–515. 33539959 10.1016/j.ijbiomac.2021.01.196

[38] Vernice NA , Berri N, Bender RJ, Dong X, Spector JA. Production of a low-cost, off-the-shelf, decellularized cartilage xenograft for tissue regeneration. Ann Plast Surg 2022;88:S296–S301. 35513335 10.1097/SAP.0000000000003185 PMC9097345

[39] Prein C , Beier F. ECM signaling in cartilage development and endochondral ossification. Curr Top Dev Biol 2019;133:25–47. 30902255 10.1016/bs.ctdb.2018.11.003

[40] Rahmati M , Nalesso G, Mobasheri A, Mozafari M. Aging and osteoarthritis: central role of the extracellular matrix. Ageing Res Rev 2017;40:20–30. 28774716 10.1016/j.arr.2017.07.004

[41] Qiu Z , Wei Y, Chen N, Jiang M, Wu J, Liao K. DNA synthesis and mitotic clonal expansion is not a required step for 3T3-L1 preadipocyte differentiation into adipocytes. J Biol Chem 2001;276:11988–95. 11278974 10.1074/jbc.M011729200

[42] Parker DC. T cell-dependent B cell activation. Annu Rev Immunol 1993;11:331–60. 8476565 10.1146/annurev.iy.11.040193.001555

[43] Theodoridis K , Manthou ME, Aggelidou E, Kritis A. In vivo cartilage regeneration with Cell-Seeded natural biomaterial scaffold implants: 15-Year study. Tissue Eng Part B Rev 2022;28:206–45. 33470169 10.1089/ten.TEB.2020.0295

[44] Hu C , Li L. Preconditioning influences mesenchymal stem cell properties in vitro and in vivo. J Cell Mol Med 2018;22:1428–42. 29392844 10.1111/jcmm.13492 PMC5824372

[45] Yang Z , Li H, Yuan Z, Fu L, Jiang S, Gao C, Wang F, Zha K, Tian G, Sun Z, Huang B, Wei F, Cao F, Sui X, Peng J, Lu S, Guo W, Liu S, Guo Q. Endogenous cell recruitment strategy for articular cartilage regeneration. Acta Biomater 2020;114:31–52. 32652223 10.1016/j.actbio.2020.07.008

[46] Dhanasekaran DN , Reddy EP. JNK-signaling: a multiplexing hub in programmed cell death. Genes Cancer 2017;8:682–94. 29234486 10.18632/genesandcancer.155 PMC5724802

[47] Guo XX , An S, Yang Y, Liu Y, Hao Q, Tang T, Xu TR. Emerging role of the jun N-terminal kinase interactome in human health. Cell Biol Int 2018;42:756–68. 29418027 10.1002/cbin.10948

[48] Gao W , Chen K, He W, Zhao S, Cui D, Tao C, Xu Y, Xiao X, Feng Q, Xia H. Synergistic chondrogenesis promotion and arthroscopic articular cartilage restoration via injectable dual-drug-loaded sulfated hyaluronic acid hydrogel for stem cell therapy. Compos B Eng 2023;263:110857.

