# Supplementary material for: Chondrogenic medium in combination with a c-Jun N-terminal kinase inhibitor mediates engineered cartilage regeneration by regulating matrix metabolism and cell proliferation
Source: Regen Biomater. 2023 Sep 7;10:rbad079. doi: 10.1093/rb/rbad079 (PMC10640392; doi:10.1093/rb/rbad079)
Supplement: rbad079_Supplementary_Data [file rbad079_supplementary_data.docx]

| **Gene Name** | **Forward**  **Primer (5’-3’)** | **Reverse**  **Primer (5’-3’)** | **Amplicon Size (bp)** |
| --- | --- | --- | --- |
| *β-actin* | GCAGAAACGAGACGAGATTG | GCAGAACTTTGGGGACTTTG | 167 |
| *ACAN* | CACCCCGAGAATCAAATGGA | TGGGCAGCGAGACCTTGT | 116 |
| *COLⅡA1* | TCCTGTGCGACGACATAATCT | GCAGTGGCGAGGTCAGTAG | 103 |
| *Sox9* | AGGTGCTCAAGGGCTACGAC | TTGACGTGGGGCTTGTTCT | 82 |
| *TNF-α* | CCTGTGCCTCCCTTCACTTAT | TTTCTCGCCACTGACCAGTAG | 157 |
| *IL-1β* | GTCTTCCTAAAGCAAGCCTTAC | GGGGTGTCACAATCTGTTTC | 92 |
| *MMP13* | CGGGAATCCTGAAGAAGAAT | AAGTTTGCCTGTCACCTCTAAG | 92 |

**Supplementary Materials**

**Supplementary Table Ⅰ.** Forward and reverse primer sequences of genes that were analyzed


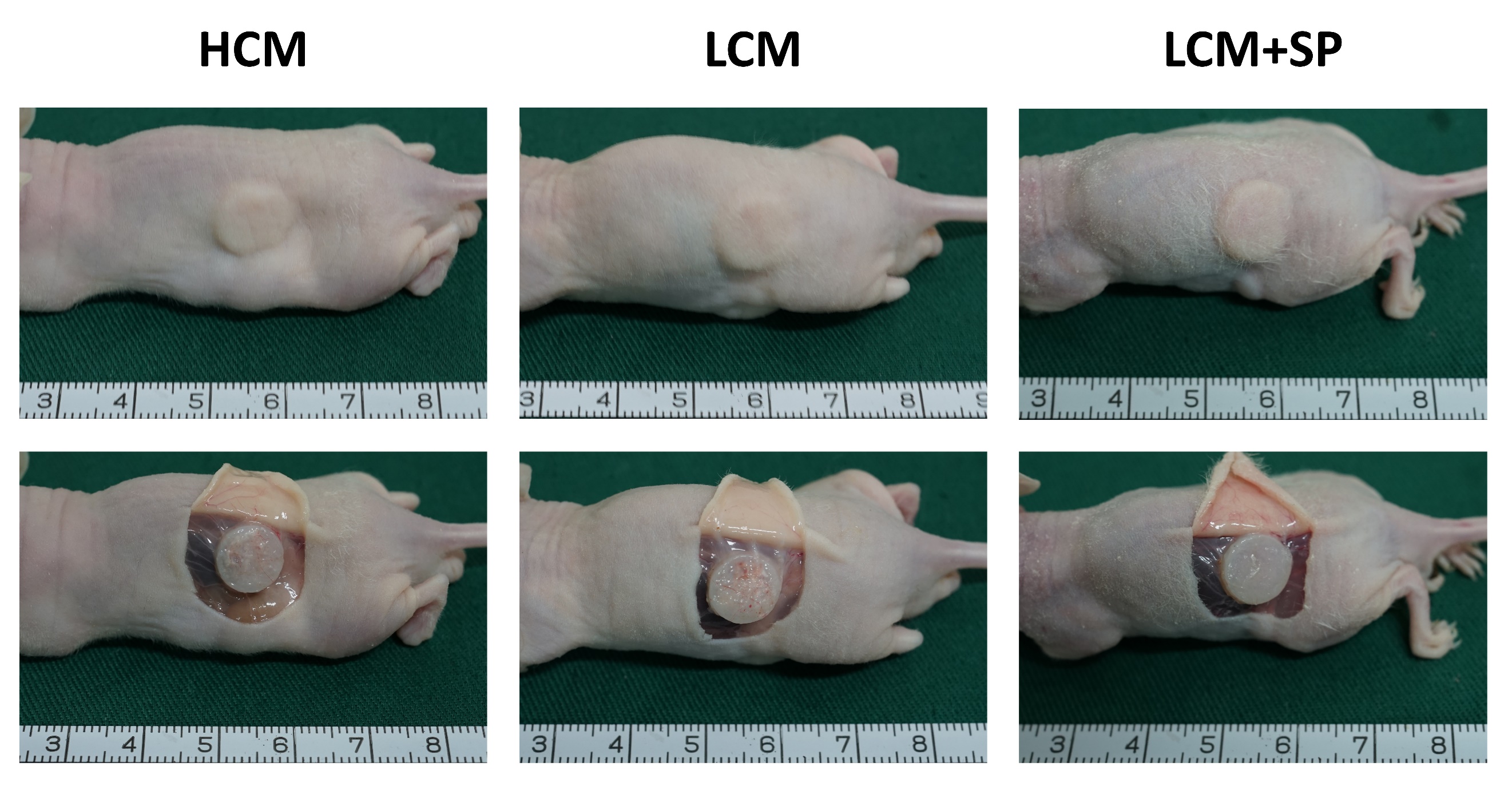


**Supplementary Figure 1.** Gross view of regenerated cartilages under the skins of nude mice after 8 weeks of *in vivo* implantation.
